# Mechanism of action of the moonlighting protein EfTu as a Substance P sensor in *Bacillus cereus*

**DOI:** 10.1038/s41598-018-37506-6

**Published:** 2019-02-04

**Authors:** Awa R. N’Diaye, Valerie Borrel, Pierre-Jean Racine, Thomas Clamens, Segolene Depayras, Olivier Maillot, Beatrice Schaack, Sylvie Chevalier, Olivier Lesouhaitier, Marc G. J. Feuilloley

**Affiliations:** 1 Laboratory of Microbiology Signals and Microenvironnement, LMSM EA 4312, Normandie Univ.; University of Rouen Normandy, 27000 Evreux, France; 2 University Grenoble Alpes, CEA, CNRS, IBS, F-38000 Grenoble, France; 3 University Grenoble Alpes, CNRS, CHU Grenoble Alpes, Grenoble INP, TIMC-IMAG The REx team, F-38000 Grenoble, France

## Abstract

The striking feature of the ubiquitous protein EfTu (Thermo unstable ribosomal Elongation factor) is its moonlighting (multifunctional) activity. Beyond its function at the ribosomal level it should be exported to the bacterial surface and act as an environmental sensor. In *Bacillus cereus*, and other cutaneous bacteria, it serves as a Substance P (SP) receptor and is essential for bacterial adaptation to the host. However, the modus operandi of EfTu as a bacterial sensor remains to be investigated. Studies realized by confocal and transmission electron microscopy revealed that, in the absence of an exogenous signal, EfTu is not exposed on the bacterial surface but is recruited under the effect of SP. In addition, SP acts as a transcriptional regulator of the *tuf* gene encoding for EfTu. As observed using gadolinium chloride, an inhibitor of membrane mechanosensitive channels (Msc), Msc control EfTu export and subsequently the bacterial response to SP both in terms of cytotoxicity and biofilm formation activity. Microscale thermophoresis revealed that in response to SP, EfTu can form homopolymers. This event should occur after EfTu export and, as shown by proteo-liposome reconstruction studies, SP appears to promote EfTu polymers association to the membrane, leading subsequently to the bacterial response. Molecular modeling suggests that this mechanism should involve EfTu unfolding and insertion into the bacterial cytoplasmic membrane, presumably through formation of homopolymers. This study is unraveling the original mechanism action of EfTu as a bacterial sensor but also reveals that this protein should have a broader role, including in eukaryotes.

## Introduction

Known essentially for its involvement in foodborne diseases, *Bacillus cereus* is seen as a member of the transient skin microflora^1^. This bacterium expresses a large arsenal of virulence factors, including hemolysins, phospholipases, emetic toxin and pore forming enterotoxins^2^, explaining at least partly its involvement in primary cutaneous infections^1,3^. However, its association with skin in the absence of any clinical sign^4^ suggests that this bacterium can also grow as a skin commensal microorganism. A strain such as *B. cereus* MFP01, isolated from normal skin^4^, also expressed virulence factors such as the emetic toxin cereulide and collagenase^5^ but this activity was strongly dependent of local host factors. Indeed, Substance P (SP), the principal skin neuropeptide, which is released in significant amounts in sweat and tissue matrix^6,7^, has a strong boosting effect on *B. cereus* virulence^5^. The tertiary structure and charge of SP, poorly compatible with transmembrane diffusion, and the speed of the bacterial response suggested that SP was interacting with a membrane sensor identified as Thermo unstable ribosomal Elongation factor EfTu^5^. EfTu was also found as a SP binding site in other Gram positive bacteria such as *Staphylococcus aureus* and *Staphylococcus epidermidis*^8^. The amount of EfTu expressed in bacteria was found to be four to fifteen times higher than required for its stoichiometric association to ribosomes and could reach up to 5% of total bacterial proteins^9^. That an organism such as a bacterium, fitting continuously its metabolism to its strict requirements, expresses in large quantities a molecule as expensive to synthesize as EfTu hinted to other functions for this molecule. Hence, EfTu is now recognized as a “moonlighting protein”, a type of multifunctional protein playing totally different roles in the bacterial stroma and on its surface^10,11^. For instance, in *Bacillus anthracis* EfTu was identified as a plasminogen receptor^12^. In *Lactobacillus johnsonii*, it is considered as an adhesin required for the pro-inflammatory response^13^ and in *Agrobacterium tumefaciens* as a Pathogen-Associated Molecular Pattern (PAMP) recognized by plants during infection^14^. In *Mycoplasma pneumoniae* and *Acinetobacter baumani*, EfTu was found as a bacterial adhesin and fibronectin binding protein^15,16^. In *Pseudomonas aeruginosa*, EfTu was identified as a binding site for Factor H and plasminogene^17^, GABA^18^ and more recently as a ligand for SST6 toxins^19^.

It has been suggested that in *Escherichia coli* EfTu could be translocated at the bacterial surface through the large mechanosensitive channel MscL^20^. However, the modus operandi of EfTu as a bacterial sensor remained to be investigated. Among the major questions still unsolved, we should quote:

- If EfTu is initially intracellular, it should be the sensor triggering its own export, but in this case what is the signal inducing this export?
- If EfTu requires a channel to be exported, and therefore appears unable to insert itself into the membrane, how can it interact with the membrane from the outside and mediate a bacterial response?
- How extracellular signals can mediate a signal leading to the bacteria response after binding to EfTu?

In the present study, we took advantage of our experience on the involvement of moonlighting proteins in the bacterial response^5,8,19,21,22^ to decipher the mechanism of action of EfTu in the response of *B. cereus* to SP. To this end, we used CLSM and TEM to localize EfTu before or after exposure to SP. The potential effect of SP on EfTu expression was quantified by qRT-PCR and an inhibitor of mechanosensitive channels (Msc) was used to investigate the effect of SP on EfTu export. In parallel, the link between EfTu export and the effect of SP on the cytotoxic and biofilm formation activities of *B. cereus* was studied. A recently developed technique, microscale thermophoresis (MST), was used to investigate the action of SP on EfTu organization. Proteoliposome reconstitution assays were carried out to document the potential interactions of EfTu and SP with membrane phospholipids. In the light of the results, a model of EfTu organization was extrapolated *in silico* and a mechanism of action of EfTu as a bacterial SP sensor was modeled for the first time.

## Results

### Substance P induces surface exposure of EfTu in *B. cereus*

**Figure 1:**
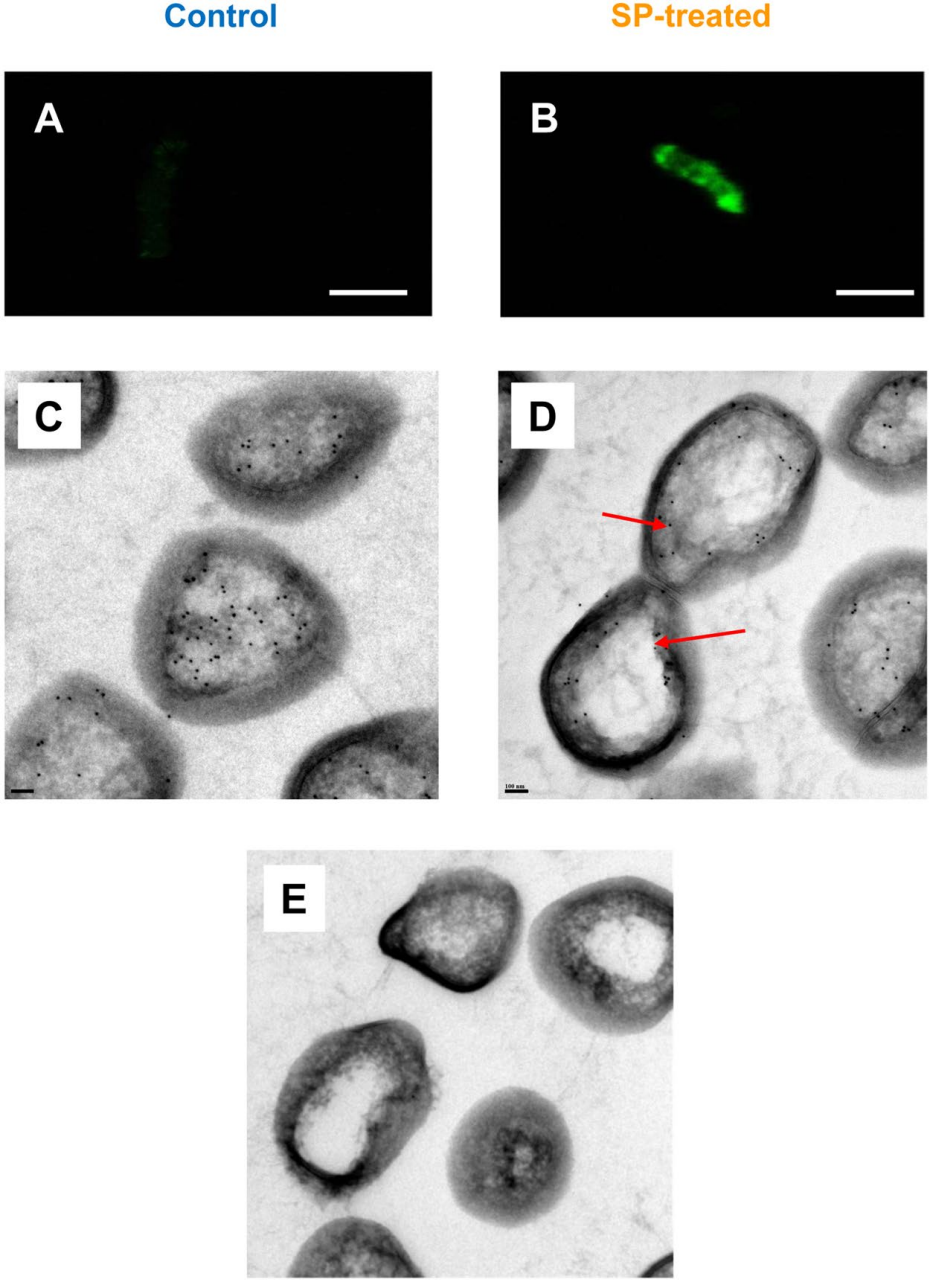
Effect of SP on the expression of immunoreactive EfTu at the surface of *B. cereus*. EfTu was localized by CLSM (**A**,**B**, Scale Bar = 2 µM) and electron microscopy (**C–E** Scale Bar = 100 nm) in control (left part) and SP treated bacteria (right part) using EfTu polyclonal antibodies. For CLSM, immunolabeling was realized in the absence of permeabilization procedure. For electron microscopy, EfTu was localized on ultrathin section by the immunogold technique. Arrows indicate EfTu immunoreactive material localized at the bacterial periphery. E: Control view realized by exposing bacteria to EfTu polyclonal antibodies preincubated with *P. aeruginosa* EfTu (10^−6^ M).

To investigate whether SP was actually modifying the distribution of EfTu into *B. cereus*, observations were realized by CLSM on intact bacteria using anti-EfTu antibodies in the absence of permeabilization treatment. In these conditions antibodies are usually unable to break through the bacterial plasma membrane and can only stain molecules and epitopes exposed on the cell surface. These studies revealed that, in the absence of exogenous signal, *B. cereus* was not presenting EfTu immunoreactive surface proteins and bacteria remained unstained (Fig. 1A). Conversely, bacteria exposed to SP (10^−6^ M) showed a strong anti-EfTu labeling (Fig. 1B). As the protocol involved a dehydratation step before the immunostaining, the bacterial structure was poorly preserved but the distribution of EfTu immunoreactivity was not homogeneous and appeared essentially localized at the periphery of the bacterium. EfTu distribution into *B. cereus* was then investigated by transmission electron microscopy by immunogold labeling using the same anti-EfTu antibody. As this technique required the realization of ultrathin sections, both surface and intra-bacterial EfTu were accessible to the labeling. In control bacteria, EfTu immunoreactivity was visualized throughout all the stroma (Fig. 1C). Conversely, in SP-treated bacteria, EfTu appeared essentially concentrated, but not exclusively, at the periphery and in the sub-membrane areas (Fig. 1D). No straining was observed when antibodies were pre-incubated with *P. aeruginosa* EfTu (10^−6^ M) (Fig. 1E).

### Substance P regulates *B. cereus* EfTu mRNA expression

**Figure 2:**
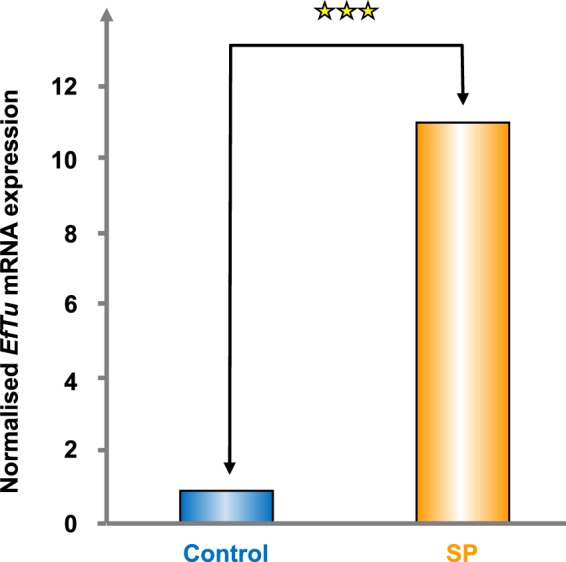
Effect of SP on *EfTu* mRNA transcription in *B. cereu*s. Expression of EfTu mRNAs was quantified by qRT-PCR using primers presented in Table 1 and a 7300 Real Time PCR System apparatus. EfTu expression was normalized using 16S mRNA as reference. ^★★★^*p* < 0.001.

EfTu mRNAs were quantified by qRT-PCR in control and SP-treated *B. cereus* using 16S rRNA as endogenous control. As shown in Fig. 2, exposure of *B. cereus* to SP (10^−6^ M) led to a highly increase of EfTu mRNAs expression (*P* value = 0.0019) that reached 10.9 fold the level measured in control bacteria indicating that the *tuf* gene encoding EfTu is submitted to a SP dependent transcriptional regulation.

### Gadolinium chloride blocks EfTu export in control and Substance P treated *B. cereus*

**Figure 3:**
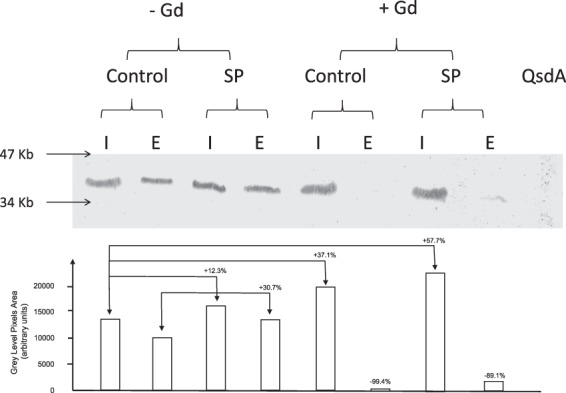
Effect of gadolinium chloride (Gd) on the intra- and extracellular concentrations of EfTu produced by control and SP treated *B. cereus*. Bacteria exposed to GdCl_3_ (1 mM) were subsequently grown in the absence or presence SP (10^−6^ M). Proteins extracted from intra-(I) and extracellular (E) compartments were analyzed by SDS-PAGE. EfTu was identified by western blot using *P. aeruginosa* EfTu polyclonal antibidodies. I: intracellular; E: extracellular. The N-acyl homoserine lactonase, QsdA, was used as a control. Results are representative of three independent experiments. Densitometric analysis data are presented as histograms.

Gadolinium chloride (GdCl_3_) was previously shown as an inhibitor of Msc targeting essentially the MscL channel^23^. The effect of GdCl_3_ on EfTu export by *B. cereus* in control conditions and after exposure to SP was investigated by western blot using antibodies raised against *P. aeruginosa* EfTu. In control bacteria, EfTu was essentially detected in the intracellular compartment although a significant amount was also found in the extracellular medium (Fig. 3). The pool of EfTu was visibly increased after exposure of the bacteria to SP (10^−6^ M). Densitometric analysis revealed a 12.3 and 30.7% increase of intra- and extracellular EfTu in SP treated bacteria, respectively. When bacteria were exposed to GdCl_3_ (1 mM), the level of intracellular EfTu in untreated and SP treated bacteria reached +37.1 and +57.7% of the control. Conversely, EfTu export was almost totally blocked in GdCl_3_ treated bacteria with a 99.4 and 89.1% decrease of extracellular EfTu, independently of the absence or presence of SP.

### Gadolinium chloride inhibits the effect of Substance P on *B. cereus* cytotoxicity and biofilm formation activity

**Figure 4:**
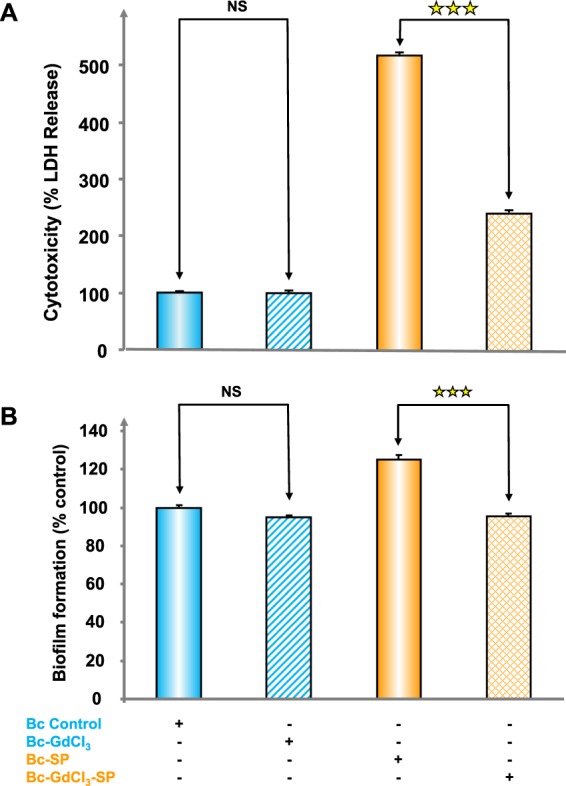
Effect of gadolinium chloride (GdCl_3_) on the cytotoxicity (**A**) and biofilm formation activity (**B**) of control and SP treated *B. cereus*. Bacteria exposed to GdCl_3_ (1 mM) were subsequently grown in the absence or presence SP (10^−6^ M). The cytotoxic potential of *B. cereus* was measured on HaCaT cells using LDH release assay after 2 h incubation. The biofilm formation activity of bacteria was observed using the crystal violet technique after 24 h incubation. Each value represents the mean ± SEM of three independent experiments. (NS: not significantly different; ^★★★^*p* < 0.001).

The potential link between EfTu export through Msc channels and the response of *B. cereus* to SP was investigated by testing the effect of GdCl_3_ on the cytotoxicity and biofilm formation activity of control and SP treated bacteria. *B. cereus* cytotoxicity was studied on HaCaT keratinocytes using a LDH release assay. The cytotoxicity of the bacterium was not modified by exposure to GdCl_3_ (1 mM) (Fig. 4A). As previously observed^5^, SP (10^−6^ M) induced a strong increase of *B. cereus* cytotoxicity (+517 ± 10%). When *B. cereus* was treated by GdCl_3_ (1 mM), the effect of SP reached 242 ± 9% of the control and was significantly reduced (−53.12 ± 0.06%, *p* < 0.001). The biofilm formation activity of *B. cereus* was analyzed after 24 h incubation in microplates using the crystal violet technique. GdCl_3_ (1 mM) alone induced a marginal and non-significant decrease of biofilm formation (Fig. 4B). Conversely, SP (10^−6^ M) stimulated the production of biofilm by *B. cereus*, its level reaching +125.1 ± 2.4% of the control. This effect of SP was totally inhibited by GdCl_3_ and the biofilm production reached the level observed when bacteria were exposed to GdCl_3_ alone.

### Substance P promotes EfTu multimerization

**Figure 5:**
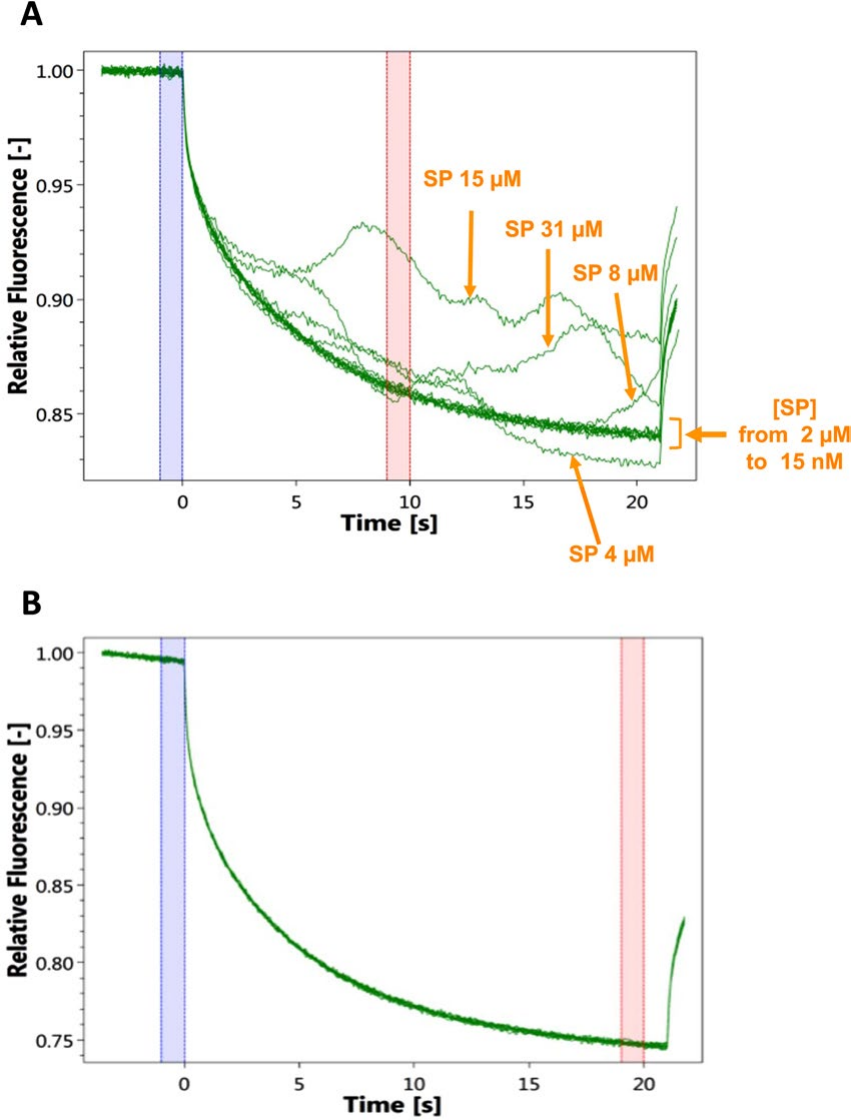
Effect of SP on EfTu oligomerization. Thermophoresis graphs show in green the evolution of the relative fluorescence of RED-NHS labeled EfTu (**A**) and AmiC (**B**) in the presence of increasing concentrations of SP. When SP 15 nm to 2 µM was interacting with EfTu no shift of the asymptotic value of the decreasing parabolic curve, characteristic of ligand-receptor interaction, was observed. At concentrations of SP from 4 µM to 31 µM association curves showed reproductive and important variations of intensity characteristic of aggregates formation of the labeled EfTu. Aggregates formation was not observed when SP was incubated in the presence of AmiC. Bleue and pink bars represent the regions where the MST signal is measured for calculation of the Kd values (value not calculable presently). The curves are representative of 3 independent experiments.

MST is a recently developed technique aimed at measuring the direct binding of two molecules, including proteins and peptides. MST interaction curves of EfTu with SP showed an unexpected difference of behaviour between 2 and 4 µM SP. For SP concentrations between 15 nm and 2 µM, the curves overlapped and no signal of interaction, characterized by a shift of the asymptotic value of the decreasing parabolic curve^24^, could be detected (Fig. 5A). Hence the K_D_ value for interaction of SP with EfTu was impossible to determine. However, at concentrations of SP above 4 µM and up to the maximal concentration tested (31 µM), association curves showed reproductive and important variations of intensity, which characteristic of aggregates formation^25^. It is not possible to determine the number of EfTu monomers involved in the formation of these multimeric forms by analysing the MST signal. However, this reaction clearly appears to be associated with the interaction of SP with EfTu as realization of the same experiments using another bacterial protein, presently the amidase AmiC, did not lead to any detectable signal variation (Fig. 5B).

### Substance P favours EfTu association to lipidic membranes

**Figure 6:**
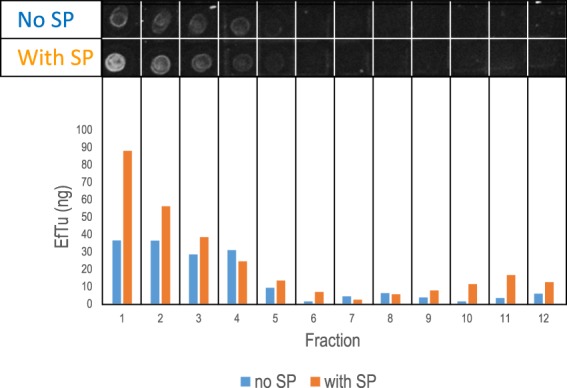
Distribution of EfTu in liposomes in response to SP. RED-NHS labelled EfTu was mixed with liposomes in the absence (blue) or presence (orange) of 20 nmoles SP. After interaction, proteoliposomes were separated by ultracentrifugation on a sucrose gradient. The gradient was split into 14 fractions and the amount of RED-NHS labelled EfTu was quantified at 670 nm using a TECAN Spark 20M spectrofluorimeter. Lipids were quantified by spectrophotometry using ANS labeling as shown in dotblots at the top of the figure. The ratio of EfTu in contact with liposomes (estimated as lipids) varied between 38 and 48 ng EfTu/mg of lipids. Because of their density, liposomes accumulated in top fractions 1 to 4. EfTu was also detected principally in these fractions. The presence of SP increased the amount of EfTu found in fraction 1 to 4 (+154.9%). This figure is representative of 3 independent experiments. Dots figure originates from a single view of all dots without individual treatment.

Geertsma *et al*.^26^. have described in detail the relipidation of membrane proteins: the purified membrane protein was first inserted into detergent-destabilized preformed liposomes and the detergent was then subsequently removed by adsorption onto polystyrene beads (biobeads). We performed EfTu relipidation studies using liposomes with membrane constitution similar to *B. cereus* native membrane (phosphatidylethanolamine PE 40%, phosphatidylglycerol PG 40%, diphosphatodylglycerol CL 20%)^27^. RED-NHS labelled EfTu was mixed with liposomes in the absence or presence of SP (20 nmoles). After interaction and rinsing, proteoliposomes were separated by ultracentrifugation on a sucrose gradient. The gradient was split into 14 fractions and the amount of RED-NHS labelled EfTu was quantified at 670 nm using a TECAN Spark 20M spectrofluorimeter. Lipids were quantified by spectrophotometry using ANS labeling, The ratio of EfTu in contact with liposomes (estimated as lipids) varied between 38 and 48 ng EfTu/mg of lipids. Because of their density, liposomes accumulated in top fractions 1 to 4 (Fig. 6). EfTu was also detected principally in these fractions. The presence of SP increased the amount of EfTu found in fraction 1 to 4 (+154.9% by comparison of the amout of RED-NHS labelled EfTu found in fractions 1 to 4 in the absence or presence of SP after correction of the protein/lipid ratio).

### Molecular model of EfTu interactions

**Figure 7:**
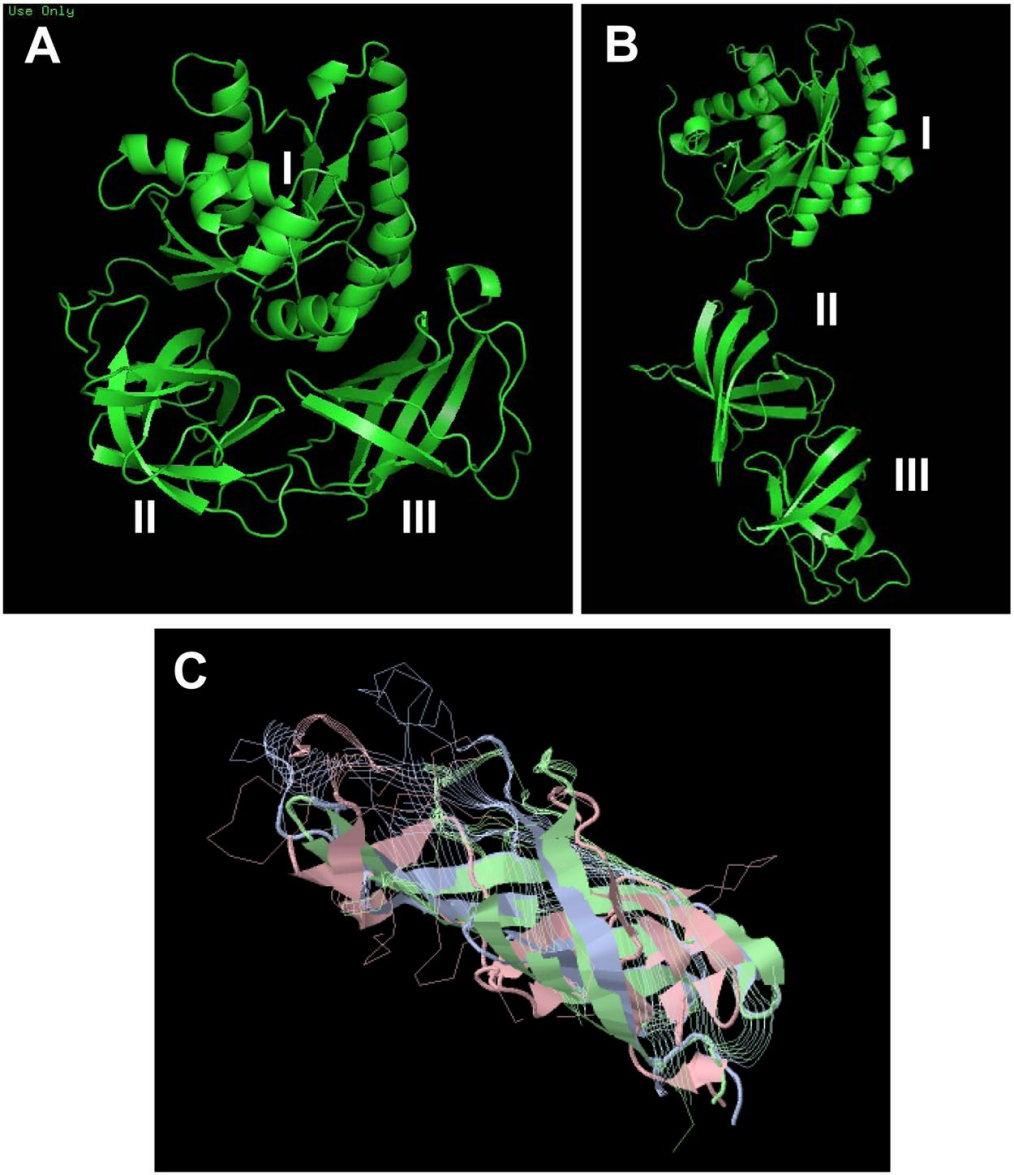
Molecular modeling of EfTu and SP interaction. A globular structure of *B. cereus* EfTu1 (UNIPROT A0A0G8DYE7), showing the typical 3 domains (I, II and III) was generated under RaptorX by alignment over *Escherichia coli* EfTu RCSB 1EFC (**A**). An unfolded structure, generated by PyMOL and submitted to CABS-flex server analysis, showed a potential rotation point between LYS296 and GLU395 (**B**). Both folded and unfolded models showed similar energy distribution (tension) and potential stability. The two hydrophobic barrel domains II and III of *B. cereus* EfTu were aligned with the trans-membrane region of OmpW and OprF using RaptorX Structure Alignment (**C**). Over the 165 to 193 amino acids involved in the transmembrane region of these porins, 83 were homologous in *B. cereus* EfTu.

**Figure 8:**
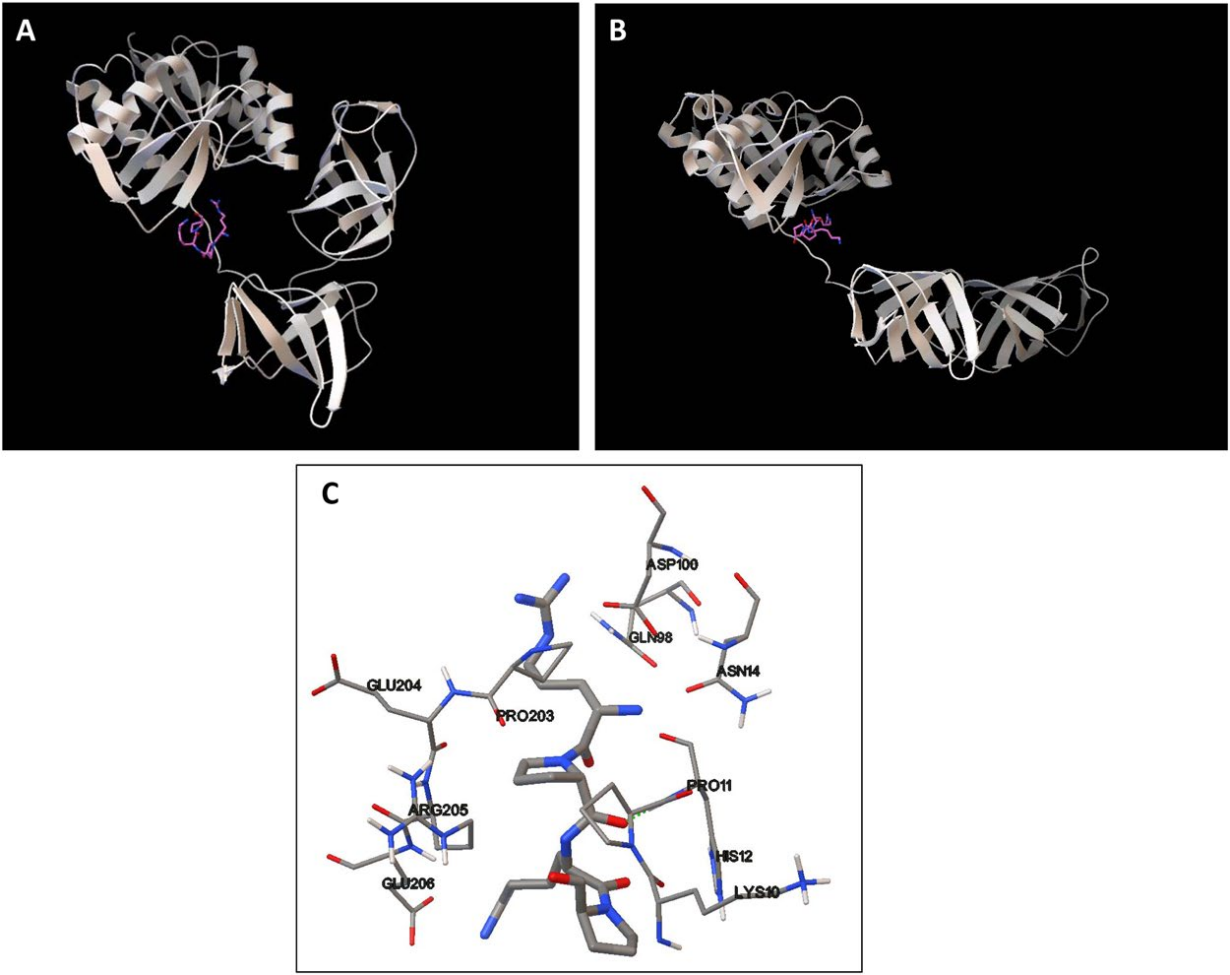
Molecular modeling of EfTu interaction with SP. Binding of SP to folded (**A**) and unfolded (**B**) *B. cereus* EfTu calculated using AutoDock 4.2 should occur at the level of the inter-region between domains I and II of EfTu and the 4 N-terminal polar amino acids of SP. In the binding region (**C**), SP (bold lines) should interact principally with amino acids LYS10 – PRO11 – HIS12 – ASN14 – GLN98 – ASP100 – PRO203 – GLU204 – ARG205 – GLU206. The center of the grid for docking was EfTu LYS90 (size 126x126x126 points, path 0.375 A). Calculated binding values between SP and folded or unfolded EfTu were −7.85 and −7.75 Kcal/mol, respectively.

On the basis of the sequence UNIPROT A0A0G8DYE7 of *B. cereus* EfTu1, a 3D model was generated under RaptorX Structure Prediction by alignment on the crystalized *E. coli* EfTu RCSB 1EFC structure (Fig. 7A). This model had the typical globular form of EfTu^21^ with domain I essentially organized in an α helix, whereas domains II and III form β strands. However, we noticed that domains II and III contained a high percentage of hydrophobic amino acids (56%) and that β strands formed short barrels. An unfolded forms of *B. cereus* EfTu obtained by rotation between LYS296 and GLU395 was calculated with PyMOL and tested using the CAB-flex server (Fig. 7B). Folded and unfolded conformations of EfTu appeared equally valuable. The two models were further analysed by EXPAZY Pro-SA to determine the potential energy distribution (tension) and the Z-Scores were of the same range (between −6.88 and −7.91 for the unfolded form and −7.59 to −8.76 for the folded form). In particular, in both models the tension into the sequence between domains I and II submitted to rotation was low. In the unfolded model of EfTu, domains II and III forming short hydrophobic barrels (17.9 to 18.3 Å and 17.7 to 24.5 Å, respectively), could be aligned. The structure formed by the association of these two domains was of the same size (35.6 to 42.8 Å) as observed in some outer membrane porins such as OmpX (36.3 to 37.8 Å, P0A917 OMPX_ECOLI), OmpW (31.4 to 51.6 Å, P0A915 OMPW_ECOLI) or OprF (27.9 to 44.5 Å, P13794 PORF_PSEAE). Moreover, superposing of these two EfTu barrels with OmpW and OprF using RaptorX Structure Alignment (Fig. 7C) revealed that over the 165 to 193 amino acids involved in the transmembrane region of these porins, 83 were homologous in *B. cereus* EfTu. Hydrophobicity profiles of OmpW, OmpX OprF and unfolded EfTu were compared using ExPASy ProtScale. The Kyte and Doolittle hydrophobicity profiles were closely resembling with in each case 4 decreases and 4 increases of hydrophobicity degree. Taken together these bioinformatics studies suggest that, in agreement with liposomes EfTu incorporation studies, an unfolded form of EfTu can insert into a membrane through interaction with the two hydrophobic barrel domains II and III. As EfTu was identified as the SP binding site^5,8^, the association of SP to both folded and unfolded EfTu configurations was studied using AutoDock 4.2. As shown in Fig. 8A,B, validated by CABS-flex, the binding should occur at the level of the inter-region between domains I and II of EfTu and the 4 N-terminal polar amino acids of SP. Other 7 amino acids of SP are hydrophobic and form a short helix. The centre of the grid for docking was EfTu Lys 90 (size 126 × 126 × 126 points, path 0.375 A). Calculated binding values between SP and folded or unfolded EfTu were −7.85 and −7.75 Kcal/mol, respectively. Principal EfTu amino acids potentially involved in interaction with SP are presented in Fig. 8C. As MST studies suggested a potential formation of EfTu homopolymers, this hypothesis was also studied by bioinformatics using GalaxyWEB GalaxyHomomer. It appeared that in its globular and unfolded forms, EfTu monomers should be capable to interact between on the one side GLN98 - MET99 - ASP100 - GLY127 – PRO129 - TYR130 - PRO201 - THR202 - GLU204 and on the other side ASP208 - PRO210 – SER297 – LYS298 - GLY298- SER299- VAL300 - LYS301 - ILE370 – GLU371. The potential interaction of these EfTu sequences reached a confidence V values between −3.48 and −4.07 sufficient to consider it as significant. Minimal potential mechanical constraints polymers appeared as octameric structure with pore-ring structure.

## Discussion

EfTu has been identified as the unique binding protein and sensor of SP in *B. cereus*^5^ but, since this protein is meant to be essentially involved in protein elongation at the ribosomal level and therefore should be present in the cytoplasm, the effect of SP should be explained either by entrance of SP into the bacterium or conversely by translocation of EfTu to the bacterial surface. It has been demonstrated that, in contact with lipidic membranes, SP does not behave as a linear peptide. Its N-terminus is hydrophilic whereas the C-terminal sequence between amino acids 5 to 11, potentially organized as α helix, should interact with the aliphatic moiety of phospholipids^28^. Therefore, SP could hardly cross the bacterial membrane. In addition, the speed of the response of *B. cereus* to SP, reaching 95% of its maximum in less than 5 min^5^, is not compatible with diffusion mechanisms, generally slow, and suggested membrane recognition of the peptide and therefore exposure of EfTu on bacterial surface. CLSM observations are in agreement with this hypothesis and demonstrate that, whereas EfTu was undetectable on the bacterial surface in control conditions, SP promoted its exposure. Electron microscopy studies confirmed an accumulation of EfTu at the periphery of SP treated bacteria and in the sub-membrane area. However, in TEM studies the whole pool of EfTu was accessible to the antibodies and a labelling of intra-cytoplasmic EfTu was also observed. Necessarily, the migration of EfTu from the bacteria cytoplasm to the membrane would require a motor system and it is interesting to note that EfTu was found associated to the actin-like protein MreB in the submembrane area of *E. coli* and *B*. subtilis^29^ and more recently that EfTu could even direct MreB polymerization^30^. As shown by qRT-PCR, in parallel to mobilizing EfTu towards the membrane, SP also exerted a transcriptional regulation on EfTu expression. This is consistent with the dual functions of this protein, as it is essential for the bacterium to preserve a pool of EfTu for its primary role in protein elongation at the ribosomal level. In agreement with this hypothesis, it was previously demonstrated that SP is not affecting the growth of *B. cereus* and therefore its protein synthesis activity^5^. The *tuf* gene is one of the most highly conserved in the bacterial genome and it is frequently duplicated in eubacteria as *tufA* and *tufB*. However, *tufB*, the duplicated gene, was lost in low GC Gram-positive bacteria such as Bacilli^31^. Therefore, we can conclude that SP exerts a positive effect on *tufA* transcription, the ancient gene common to all eubacteria suggesting that, as demonstrated in *Staphylococci*^8^, EfTu should act as an environmental sensor in many other bacterial species. The present results are coherent with studies showing that EfTu is a moonlighting protein that can migrate from the cytoplasm to the bacterial surface^10,11^, but to our knowledge, it is the first time that it was demonstrated that the association of EfTu with the bacterial membrane could be induced in response to an exogenous signal, *i.e*. SP.

Because of its size, 396 amino acids in *B. cereus*, the transfer of EfTu to the bacterial surface would require an export system to cross the bacterial membrane. In 2000, Berrier *et al*.^20^. suggested that, under osmotic shock, EfTu would be exported through *E. coli* membrane by large mechanosensitive channels (MscL). Mechanosensitive channels have been well studied in prokaryotes, primarily in *E. coli* and are classified into three classes, namely large (MscL), small (MscS and MscK) and minimal (MscM) conductance channels^32^. These three types of channels differ not only in their conductance, but also in their gating kinetics, sensitivity to membrane tension and three-dimensional structures. However compensation mechanisms exist between these channels and for that reason knockdown techniques are not particularly adapted to investigate their functions. In the present study, we preferred to employ gadolinium chloride (GdCl_3_), a specific inhibitor of bacterial mechanosensitive channels, particularly efficient on MscL^23^. As shown by direct assay of EfTu in the cytoplasm of *B. cereus* and in the extracellular medium, GdCl_3_ induced an inhibition of EfTu export by control and SP treated bacteria. These results indicate clearly that EfTu export is a mechanism totally dependent of active mechanosensitive channels, presumably MscL, and that, extracellular EfTu does not mainly result from the spontaneous lysis of a subset of the bacterial population, as commonly assumed. Moreover, GdCl_3_ inhibited the increase of cytotoxicity and biofilm formation induced by SP in *B. cereus* showing that functional mechanosensitive channels are required for the response of *B. cereus* to SP.

Western blot studies also clearly showed that in control conditions, EfTu could be detected both in the intra- and extracellular compartments indicating that the protein was continuously exported. SP increased intracellular EfTu by 12% and its export by 30%. This may seem small in comparison to the 10 fold increase in mRNA expression but we must take into consideration the dilution factor within the extracellular environment. However, western blot results appeared to be in contradiction with CLSM observations showing a total absence of EfTu at the surface of untreated bacteria. Nevertheless, it is important to note that only proteins tightly associated with the membrane surface can be visualized by CLSM, as rinsing steps discard soluble or weakly bound molecules, whereas western blot studies used extracellular extracts, therefore containing soluble proteins. So far, the only hypothesis deduced from these results was that the binding of EfTu to the extracellular layer of the plasma membrane required SP.

In order to investigate this hypothesis, we used microscale thermophoresis (MST), a technique allowing the study of the direct association of proteins in solution^24^. At concentrations lower than 2 µM, no binding of SP to EfTu was observed, whereas the threshold of SP on *B. cereus* activity was nanomolar^5^. However, at SP concentrations above 4 µM, the MST signal revealed the formation of EfTu aggregates and this effect appeared specific to the interaction of SP with EfTu since it was not observed with another and unrelated protein, the amidase AmiC. Because of the denaturation step used to recover SP-bound EfTu by the technique employed by Mijouin *et al*.^5^, it was impossible to know whether EfTu was initially in monomeric or multimeric forms when it was associated to SP. However, in the former study, the initial interaction of SP occurred in the living bacterium, *i.e*. in the complex membrane environment, whereas for MST, the interaction of SP with EfTu was promoted in an artificial buffer in the absence of lipids and other membrane proteins and, in these conditions, SP would require higher concentrations to promote EfTu multimerization or the contribution of unknown chaperones. Nevertheless, it has been previously demonstrated that at high concentrations EtTu can high molecular weight polymers^9^.

The potential insertion of EfTu in membranes and the effect of SP on this process were investigated using liposomes formed on the basis of the principal phospholipids present in the *B. cereus* cytoplasmic membrane^27^. EfTu was found to be associated with the first fractions of the sucrose gradient where liposomes where also accumulating after ultracentrifugation. Moreover, as hypothesized, SP increased the association of EfTu with liposomes, suggesting that the peptide would stabilize and/or anchor EfTu within the lipid bilayer. These results are consistent with microscopic studies and western blot experiments, except that in the absence of SP no association with EfTu was detected. This difference could be explained either by the difficulty to visualize the lower amount of EfTu associated with the membrane in control conditions, the existence of accessory proteins in the membrane of living bacteria inhibiting the binding of EfTu in its native form and/or by differences of conformational changes and membrane affinity of EfTu. Indeed, as previously suggested by Dallo *et al*.^15^, a key point in the association of EfTu to the membrane should be the occurrence of a conformational change. Molecular modeling revealed that EfTu, which is normally presented as a globular protein, could unfold generating a more elongated structure. If such a configuration change was initiated before EfTu was exported, it could also help its translocation through MscL. It was found that SP was able of binding at the basis of the neck between the EfTu polar head (domain I) and the first hydrophobic domains II. In this configuration, domains II and III are aligned and the size of the resulting barrel is the same as in outer membrane porins, such as OmpW, OmpX or OprF. Moreover, as observed by bioinformatics study, there is a high sequence homology with EfTu domains II and III and the transmembrane domains of porins suggesting that the molecule could easily insert into a membrane bilayer as observed during proteoliposome formation studies. In agreement with MST experiments, *in silico* studies even suggested that EfTu could stabilize in the membrane as a multimeric pore-like structure. Because of their size, EfTu polymers should be unable to cross the bacterial wall and S-layer. Indeed, mechanisms required for macro-molecules and hyperstructures to cross the peptidoglycan wall of Gram-positive bacteria remain hypothetical but appear very limitative^33^. Moreover, although pores of 2.5 to 3.5 nm diameter, allowing the passage of small peptides such as SP have been observed in the S-layer^34^, they should represent a barrier for the passage of EfTu polymers. For those reasons, we hypothesize that the interaction of SP with EfTu should occur in the periplasmic area.

Taken together, these results allow to design a functional mechanism of action of SP in *B. cereus* through interaction with EfTu. Bacteria need to adapt to an almost unlimited number of signals but their genome can only encode a limited number of proteins. However, during their very long evolution, they developed an extreme protein fitting mechanism that lead to the production of moonlighting proteins. Some of them, such as EfTu, would be exported in the microenvironment and act as environmental sensor. Hence EfTu should be exported through the membrane by MscL. In the absence of an exogenous ligand, EfTu would remain soluble, presumably monomeric, and unable to associate with the bacterial membrane. The binding of SP to EfTu should induc conformational changes, presumably multimerization, and finally interaction with the membrane. The following transduction process leading to the bacterial response remains to be investigated but the potential for EfTu to form pore-like structures suggests that EfTu would promote a bacterial response in this way. With this in mind, it is interesting to note that high concentrations of SP have been shown to exert antimicrobial properties through the formation of pore-like structure^35^ whereas, as discussed herein, this peptide is unable to cross the plasma membrane by itself but could generate pores through interaction with EfTu. Finally, it should be noticed that EfTu is also expressed by eukaryotic cells, namely at the mitochondrial level, and that in eukaryotes questions regarding the mechanism of action of SP are still open as SP and Neurokinin receptors have limited specificity^36^ and we can hypothesize unknown function of EfTu as accessory receptor.

## Methods

### Bacterial strains and growth conditions

*Bacillus cereus* MFP01 was obtained by swabbing the skin of a human donor^4^. This strain was characterized using API® strips, 16S ribosomal RNA gene sequencing and whole proteome analysis by MALDI mass spectrometry and Biotyper^(R)^ analysis. Bacteria were grown at 37 °C in Luria-Bertani (LB). For these studies, bacteria collected at the end of the exponential growth phase were diluted in fresh broth. Gadolinium chloride (GdCl_3_) (1 mM in sterile physiological water - NaCl 0.9%), or an equivalent volume of NaCl 0.9% was added at the onset of the culture. Bacteria were grown until the beginning of the log growth phase (2 h) and were harvested by centrifugation (7,000 × *g*) or subsequently exposed to Substance P (10^−6^ M in LB) until they reached the early stationary phase. Control studies were realized by exposing bacteria to the reversed sequence peptide of SP that was shown to have no effect on *B. cereus* growth, virulence and biofilm formation activity^5^. Before use, bacteria were washed 3 times with sterile physiological water to remove any trace of free peptide. The bacterial density and the absence of contamination were controlled by plating. The viability of the bacteria under the different culture conditions was controlled in preliminary studies (data not shown). Substance P (SP) and the reversed sequence peptide (SPrev) were obtained from Polypeptides (Strasbourg, France). Gadolinium chloride (GdCl_3_) was obtained from Sigma-Aldrich (Saint-Quentin Fallavier, France).

### EfTu and EfTu antibodies

All experiments were realized using the full 397 amino acids *Pseudomonas aeruginosa* EfTu (UniProtKB/Swiss-Prot: P09591). As previously described^8^, the protein was produced by cloning in *Escherichia coli* by use of a synthetic optimized codon (MWG Operon) containing the leader sequence MHHHHHHSSGVDLGTENLYFQ*S to provide a cleavable 6*His tag. This was cloned into the NcoI and HindIII restriction sites of the pET-Duet1 vector (Novagen) and transformed into E. coli Rosetta2 (DE3) (Novagen). Proteins from mid-log growth phase bacteria were purified at 4°C using a 1 mL HisTrap crude FF column and a Superdex 200 16/600 hr column (GE Healthcare) associated to an ÄKTAxpress system (GE Healthcare). The protein was characterized by Matrix Assisted Laser Desorption Ionization Time-of-Flight mass spectrometry (MALDI-TOF/TOF) using an AutoFlex III mass spectrometer (Bruker Daltonics). MS peak list was generated using the FlexAnalysis software and submitted for peptide mass fingerprinting using the integrated software Biotools (Version 3.2). The NCBI data base was searched using the online MASCOT software and statistical sequences analyses were realized using the probability-based Mowse score. EfTu polyclonal antibodies were produced in rabbit by Biogalenys SAS (Miserey, France) by immunisation against the *Pseudomonas aeruginosa* EfTu protein. The protein was used without coupling to an epitope and injected in the presence of Freund’s adjuvant (Sigma-Aldrich). Specificity of the antiserum was controlled by western blot analysis over a total *B. cereus* MFP01 protein extract. The unique detected ligand (EfTu) was identified by MALDI TOF/TOF analysis^5^.

### Confocal laser scanning microscopy (CLSM)

Whole bacteria previously exposed to SP or SPrev (used as a control) and adsorbed on glass slides were fixed in 4% paraformaldehyde and 0.1% glutaraldehyde in PBS for 1h in ice. After rinsing with phosphate buffered saline (PBS) they were incubated with rabbit *P. aeruginosa* EfTu antibodies.EfTu. Primary antibodies were diluted to 1:100 in PBS and incubated at room temperature for 1 h. After rinsing in PBS, slides were incubated with Alexa 488-conjugated goat anti-rabbit IgG (ThermoFisher Scientific, Illkirch, France) at room temperature for 1 h. All incubation steps were realized in the absence of permeabilization buffer or adjuvant to avoid penetration of the bacterial cytoplasm by the antibodies. Specificity of the labelling was controlled by preabsorption of the EfTu antibodies with *P. aeruginosa* EfTu (10^−6^ M). Slides were examined under a confocal laser scanning microscope (CLSM 710 confocal laser-scanning microscope, Zeiss, Jena, Germany).

### Transmission electron microscopy

Control and SP-treated bacteria were fixed using 4% paraformaldehyde and 0.1% glutaraldehyde in PBS for 1 h in ice, rinsed and then embedded in gelatine. The samples were cut into small cubes (1 mM^3^) and cryo-protected by incubation in 2.3 M sucrose in 0.1 M sodium phosphate buffer pH 7.4 during 4 h at 4 °C. For cryo-sectioning, pins with frozen cubes containing bacteria were mounted on a cryo-ultramicrotome. Bacterial blocks were trimmed at −90 °C with a cryotrim-diamond knife (Diatome, Biel, Switzerland) and ultrathin cryo-sections, 80 nm thick, were cut at −110 °C with an immune-diamond knife (Diatome). Sections were picked up with a drop of 2.3 M sucrose warmed to room temperature, and transferred onto a Formvar film-coated, carbon-stabilised 100 mesh copper finder grid (Electron Microscopy Sciences, Hatfield, PA, USA). First, the grids were washed 5 times in PBS for 2 min each, then incubated for 30 min in PBS containing 1% BSA (Aurion, Wageningen, The Netherlands) as a blocking step. Sections were floated for 1 h on a drop of primary antibody, rabbit EfTu antibodies 1:100 in PBS containing 1% BSA. After washing with 0.1% BSA in PBS, the samples were incubated for 1.5 h with 10 nm gold-conjugated secondary goat anti-rabbit antibodies (Aurion) diluted to 1:40 in 1% BSA/PBS. Ultrathin cryo-sections were then fixed in 1% glutaraldehyde in PBS for 5 min to further stabilise them, followed by 6 washes in distilled water, 2 min each. Finally, the cryo-sections were embedded in a thin film of methylcellulose containing 0.4% uranyl acetate and air-dried for 5 min. The grids were mounted on an electron microscopy sample holder and inserted into G Orptis 11 Camera Opius 400 Gatan scanning transmission electron microscopy. Specificity of the labelling was controlled by preabsorption of the EfTu antibodies with *P. aeruginosa* EfTu (10^−6^ M).

### Quantitative reverse transcription-PCR (qRT-PCR)

Expression of EfTu mRNAs was quantified by qRT-PCR using a protocol adapted from Guyard-Nicodème *et al*.^37^. Briefly, two volumes of RNAprotect bacteria reagent (Qiagen, Hilden, Germany) were added to 5.10^9^ bacteria collected in early stationary phase. RNAs were extracted with the RNeasy Midi Kit and RNase-Free DNase Set (Qiagen). Residual DNAs were eliminated by acid phenol treatment. The absence of DNA was confirmed by verifying that PCR reactions failed without prior cDNA synthesis. RNAs were non-specifically converted to single-stranded cDNAs using the High Capacity cDNA Archive Kit (Applied Biosystems). mRNAs of interest were quantified by real-time PCR amplification of their cDNAs. Primers (Table 1) were designed with Primer Express 3 software in order to have a Tm between 58 to 60 °C. Each primer pair was validated by verifying that the PCR efficiency E was above 0.95, and that a single PCR product was obtained with each expected Tm. PCR reactions were performed in triplicate with the 7300 Real Time PCR System apparatus (Applied Biosystems, Illkirch, France). The 25 μl reactions contained 12.5 μl of SYBR Green PCR Master Mix (including AmpliTaq Gold DNA Polymerase, Applied Biosystems), 900 μM of each primer, and cDNAs generated from 0.01 ng of total RNA. The conditions were 95 °C for 10 min for polymerase activation, and 40 cycles at 95 and 60 °C for 60 and 30 s, respectively. ROX dye was used as passive reference to normalize for non-PCR related fluorescence variations. The relative quantification of EfTu mRNAs was obtained by the comparative Ct (2^−ΔΔCT^) method^38^, using 16S rRNA as endogenous control.

**Table 1:** Primer sets used in the present study.

bc.EfTu.for	GGTACAATCGGCCACGTTG
bc.EfTu.rev	CGATTTGATCGTATCCGCGTG
bc.gyraseA.for	CGCAATGAGTGTTATCG
bc.gyraseA.rev	CGCATATAAAACCCTACG

### EfTu western blot quantitation

Cultures of *B. cereus* were exposed to SPrev (control) or SP as previously described^5^. Extracellular extracts were obtained by centrifugation at 7,000 × *g* for 10 min and filtration on 0.22 µM disposable filter units. Intracellular extracts were obtained by solubilizing the bacteria in 6 mL of non-denaturing lysis buffer (Tris-HCl 50 mM pH 8, EDTA 4 mM, NaCl 137 mM, glycerol 10% (v/v), Triton X-100 1% (v/v), Phenylmethylsulfonyl-fluoride 1mM) and supplemented with a protease inhibitors cocktail (Boehringer, Reims, France). Bacterial lysis was completed by sonication using short pulses (1 min) on ice. The cell lysate was centrifuged at 13,000 × *g* at 4 °C for 10 min to remove unbroken cells. For both extracellular and intracellular extracts, proteins were precipitated by 10% trichloroacetic acid (TCA, v/v) overnight on ice and subsequently harvested by centrifugation at 13,000 × *g* for 20 min at 4 °C. Extracted proteins were washed three times in cold acetone, dried for 1 h at room temperature and then dissolved in rehydration buffer in a final volume of 350 μL as previously described^39^. The protein concentration was determined by Bradford assay. Proteins were separated on 12% w/v polyacrylamide 1D gel SDS-PAGE. For Western-Blot, SDS-PAGE separated proteins were transferred onto nitrocellulose membranes at 50 mA for 1h in TRIS base (192 mM) transfer buffer containing glycine (14.5% g/L w/v) and methanol (20% v/v) using a BioRad Mini TransBlot Electrophoretic Transfer Cell system. Membranes were then air-dried and immersed for 2 h in blocking buffer (Tris buffer saline (TBS): 50 mM, 150 mM NaCl, 5% whole milk). Membranes were incubated with primary antibody raised against *Pseudomonas aeruginosa* EfTu^8^ 1:100 in blocking buffer at room temperature for 2 h while shaking. After incubation, the blot was washed 3 times in 1x TBS supplemented with 0.05% (w/v) Tween 20 for 30 min and incubated for 1.5 h while shaking with secondary antibody diluted to 1/5,000 (goat anti-rabbit IgG alkaline phosphatase conjugate, Bio-Rad, Marnes-la-Coquette, France). Electrophoretic bands were detected using an alkaline phosphatase conjugate substrate kit (Biorad). Pure *P. aeruginosa* EfTu was used as positive control, and the 35.5 kDa protein *Rhodococcus eythropolis* QsdA^39^, was used as negative control. The density of immunoreactive bands was analyzed by the ImageQuant TL software.

### Cytotoxicity studies

The effect of GdCl_3_ on the cytotoxicity of control and SP treated bacteria was studied using the HaCaT keratinocytes cell line (Cell Line Services, Eppelheim, Germany). Cells grown at 37 °C in 5% CO_2_ atmosphere in Dulbecco’s modified Eagle’s medium (DMEM, Lonza) were starved of antibiotics 24 h before infection assays. Cells were used between passages 41 and 65. Cell death was quantified by measurement of lactate dehydrogenase (LDH) using the Cytotox 96 assay (Promega, Charbonnieres, France).

### Biofilm formation studies

After an overnight preculture in LB at 37 °C, *B. cereus* MFP01 was inoculated at OD_580_ = 0.08 in LB medium and subcultured for 2 h in the absence or presence of GdCl_3_ (1 mM). SP (1 µM final concentration) was added, and bacteria were grown for an additional 1 h. Bacteria were then washed and adjusted to an OD_580_ of 0.1 in 0.9% NaCl supplemented or not with GdCl_3_ and/or SP (1 µM). The bacterial suspensions were then used to study biofilm formation under static conditions at 37 °C in 96-well plates. Each well was filled with 100 μL of inoculum in LB broth and then incubated at 37 °C. After 24 h, the culture medium of each well was removed, and washed three times with 200 μL of sterile phosphate-buffered saline. The biofilm formed in each well was stained with 100 μL of 1% crystal violet solution. After 10 min of staining, each well was washed three times with deionized water (10 min per wash). After the last wash, the plate was air dried for 30 min. 100 μL of 95% ethanol was added to each well to dissolve its biofilm. The optical density (OD) of each well at 560 nm was recorded using a microplate spectrophotometer. Each assay was performed in triplicate.

### Microscale thermophoresis

Bacterial EfTu was labeled using the RED-NHS Labeling kit (NanoTemper Technologies, Munich, Germany). The labeling reaction was performed according to the manufacturer’s instructions in the supplied labeling buffer by using EfTu 20 μM at a molar dye:protein ratio ≈ 2:1 and over an incubation time of 30 min. Unreacted dye was removed with the supplied dye removal columns equilibrated with MST buffer (50 mM Tris-HCl pH 7.5, 150 mM NaCl, 10 mM MgCl_2_). The label/protein ratio was determined by photometry at 650 and 280 nm. Typically, a ratio of 0.8 was achieved.

EfTu or AmiC were respectively adjusted to 8 nm and 62 nm with MST buffer (50 mM Tris-HCl pH 7.5, 150 mM NaCl, 10 mM MgCl_2_) supplemented with 0.05% Tween-20. SP was dissolved in MST buffer supplemented with 0.05% Tween-20. A series of twofold dilutions was prepared in the same buffer to obtain SP concentrations ranging from 31 nm to 1 mM. Each SP dilution was then mixed with one volume of EfTu or AmiC dilution, leading to a final protein concentration of 4 and 31 nm, respectively, and final ligand concentrations of half the ranges above, *i.e*. 15.5 nm to 0.5 mM. After 5 min incubation, followed by centrifugation at 10,000 ×*g* for 10 min, approximately 4 μL of each solution was filled into Monolith NT Premium coated Capillaries K005 (NanoTemper Technologies GmbH, Munich, Germany). Thermophoresis was measured using a Monolith NT.115Pico instrument (NanoTemper Technologies GmbH) at an ambient temperature of 25 °C with 5 s/30 s/5 s laser off/on/off times, respectively. Instrument parameters were adjusted to 20% LED power and medium MST power. Data of three independently pipetted measurements were analyzed (NT.Analysis software version 1.5.41, NanoTemper Technologies) using the signal from Thermophoresis + T-Jump. The data were fitted using GraphPad Prism version 5, and thermophoresis figures were directly generated using this software.

### EfTu insertion into liposomes

Pure lipids (1,2-dioleoyl-sn-glycero-3-phosphoethanolamine PE, 1,2-dioleoyl-sn-glycero-3-phosphoglycerol PG and 1′,3′-bis[1,2-dioleoyl-sn-glycero-3-phospho]-sn-glycerol = cardiolipin CL) were purchased as chloroform solutions (Avanti Polar Lipids, Alabaster, USA). For liposome preparation, the desired amount of lipids (20 mg total): 40% PE, 40% PG, 20% CL, was transferred to a glass vessel and the chloroform was evaporated under nitrogen flux in a fume hood. Residual chloroform was then discarded under vacuum in a freeze dryer under 0.055 mbar atmosphere at −55 °C for 12 hrs. Dried lipids were suspended as multi-lamellar vesicles to a final concentration of 20 mg/mL in Tris-HCl 50 mM pH 7.4, NaCl 150 mM, MgCl2 10 mM. A suspension of large unilamellar vesicles (LUVs) was obtained by extrusion, successively using 200 nm and 100 nm nucleopore polycarbonate membranes (Sigma-Aldrich, Saint-Quentin Fallavier, France), mounted between two semi-chambers (Avanti Polar Lipids) in accordance with the manufacturer’s instructions. Monomodal suspension of LUVs was obtained with a mean hydrodynamic diameter of 120 nm measured by dynamic light scattering analysis (Wyatt, Santa Barbara, USA). Polydispersity was typically 15%.

Liposomes were first destabilized by adding 0.6 mM Triton X100 (Sigma-Aldrich). This detergent concentration yielded an isotropic solution of mixed phospholipid-detergent micelles. Protein insertion in the liposomes-Triton X100 was then obtained by adding EfTu for 1 hour at 21 °C in the absence and in the presence of SP. As described by Geertsma *et al*.^26^. a protein/lipid ratio of 1/20,000 mol/mol) was chosen. In the present study, the actual ratio of EfTu in contact with liposomes (estimated as lipids) varied between 38 and 48 ng EfTu/mg of lipids. EfTu-RED-NHS-tween 20 was mixed with liposomes in 0.5 mL of buffer Tris-HCl 50mM pH 7.4, NaCl 150 mM, MgCl2 10mM in Eppendorf tubes in the presence or the absence of 20 nmoles SP. Detergents (Triton X100 and Tween 20) were removed by four successive additions of 100 mg of biobeads (Biorad, Marnes la Coquette, France) to the 0.5 mL protein/lipid/detergent mixture. During detergent removal, the proteins spontaneously associated with lipids to form proteoliposomes.

Proteoliposomes formed by EfTu with and without SP were purified on a sucrose gradient in order to quantify protein insertion. We used a 10 mL discontinuous sucrose gradient (10 to 40% sucrose in buffer Tris-HCl 50mM pH 7.4, NaCl 150 mM, MgCl_2_ 10mM). The proteoliposome solution obtained after detergent removal was loaded on top of the gradient. This gradient was centrifuged for 15 h at 200 000 × *g* (70Ti rotor, Beckman Optima I70) at 4 °C. After centrifugation, the 10 mL gradient was split into 14 fractions (700 µL each) and the amount of recombinant EfTu-RED-NHS of each fraction was assessed by fluorescence spectrophotometry (TECAN Spark 20M, fluorescence excitation wavelength 620 ± 20 nm, emission wavelength 670 ± 20 nm, gain 200). Lipid concentration was measured using 8-Anilino-1-naphthalenesulfonic acid (ANS, Sigma USA) fluorescent dye. A calibration curve (from 0 to 0.5 mg/mL) of aliquots of the liposome was first carried out using a 1/500 dilution of a 0.23% ANS stock (fluorescence Excitation wavelength 310 ± 8 nm, Emission wavelength 460 ± 8 nm, gain 2,000) as explained in Cortes *et al*.^40^. Then the same 1/500 ANS dilution was applied to the EfTu-liposome and lipid concentration was deduced from the fluorescent calibration curve. The fluorescence was quantified using a black, 96-well Greiner microtiter plate (Dominique Dutscher, France) and a Clariostar spectrophotometer (BMG, USA).

### Molecular modeling

All calculations were realized using a DELL PowerEdge T420 computer equipped with 4 hard disks (4 To each leading to 12 To under RAID5). The FASTA amino acid sequence of the *B. cereus* EfTu was obtained from UniprotKB (A0A0G8DYE7_BACCE). Its 3D structure was calculated by alignment over *Escherichia coli* EfTu RCSB 1EFC^41^. The 3D structure of OprF, OmpX and OmpW was generated the same way using as pattern the RCSB files 4RLC, 2M06 and 2F1T. RaptorX Structure Prediction^42^ was used to predict proteins structures and visualized using Python Molecular Viewer V1.5.6. Folded and unfoled EfTu structures generated by PyMOL^43^ were submitted to CABS-flex server analysis. Energy distribution (tension) into the two forms was further calculated using EXPAZY Pro-SA. Alignment of the two EfTu barrels with the trans-membrane region of OmpW and OprF was realized using RaptorX Structure Alignment^44,45^. The hydrophobicity profile of the transmembrane region of OmpX, OmpW and OprF was compared to that the aligned two barrels region of unfoded EfTu using ExPASy ProtScale and the hydrophobicity profiles were calculated according to Kyte and Doolittle^46^. Docking studies were realized using AutoDock 4.2^47^ and PDB files of both folded and unfolded *B. cereus* EfTU structures. Water was subtracted and after hydrogens addition polar hydrogens were merged and gasteiger charges were added. Results were generated using the Lamarkian Genetic Algorithm of AutoDock 4.2. The formation of EfTu homopolymers was studied using GalaxyWEB GalaxyHomomer^48,49^.

### Statistical analysis

All values were calculated over a minimum of 3 independent experiments. Except for MST graphs which were analyzed using GraphPad Prism version 5 and an internal software, the non-parametric Mann-Whitney test was used to compare the means within the same set of experiments.
